# AmesNet: A Task-Conditioned Deep Learning Model with Enhanced Sensitivity and Generalization in Ames Mutagenicity Prediction

**DOI:** 10.1021/acs.chemrestox.6c00082

**Published:** 2026-06-29

**Authors:** Tyler J. Umansky, Virgil A. Woods, Sean M. Russell, Daniel J. Haders

**Affiliations:** 701335 Model Medicines, San Diego, California 92122, United States

## Abstract

Regulatory agencies require comprehensive genotoxicity assessments for novel small-molecule therapeutics prior to human trials. Developers often delay these studies until a candidate is nearing regulatory submission because they are expensive and secondary to bioactivity. This timing creates a bottleneck where late-stage failures can jeopardize >$10 million in capital and multiple years of developmental progress per candidate. The Ames assay detects a molecule’s mutagenic potential. Regulators now explicitly support the use of in silico Ames mutagenicity models through enabling legislation, dedicated FDA AI toxicology programs, internationally harmonized guidelines, and benchmark challenges. However, current Ames models suffer from a sensitivity drop-off when they evaluate molecules outside their training domain. The out-of-domain (OOD) test set from the Second Ames/QSAR International Challenge Project yielded a participant average sensitivity of 0.46. Sensitivity is the most important metric in Ames prediction because false negatives allow mutagenic compounds to advance undetected and trigger the most costly late-stage failures. Attempts to fix this sensitivity drop-off often reduce overall model performance, which can be represented by balanced accuracy (BA). For example, DeepAmes reports high levels of sensitivity (0.87) by sacrificing its BA (0.52). We introduce AmesNet, a task-conditioned Ames model that achieves class-leading performance in novel chemical spaces. AmesNet utilizes a dual branch architecture containing a molecular encoder and a dedicated channel to condition Ames assay context such as metabolic activation and bacterial strain type. In comparative benchmarks, AmesNet reached a sensitivity of 0.72 (95% CI: 0.68–0.76) and a simultaneous BA of 0.81 (95% CI: 0.78–0.83) on the OOD test data. This performance improves discrimination without the sensitivity trade-off limiting existing approaches. Structural analysis demonstrates that AmesNet recovers difficult-to-detect mutagenic compounds missed by existing models. This framework provides a high-confidence filtering mechanism that enables drug developers to turn a costly late-stage safety bottleneck into an earlier-stage mutagenicity triage.

## Graphical abstract

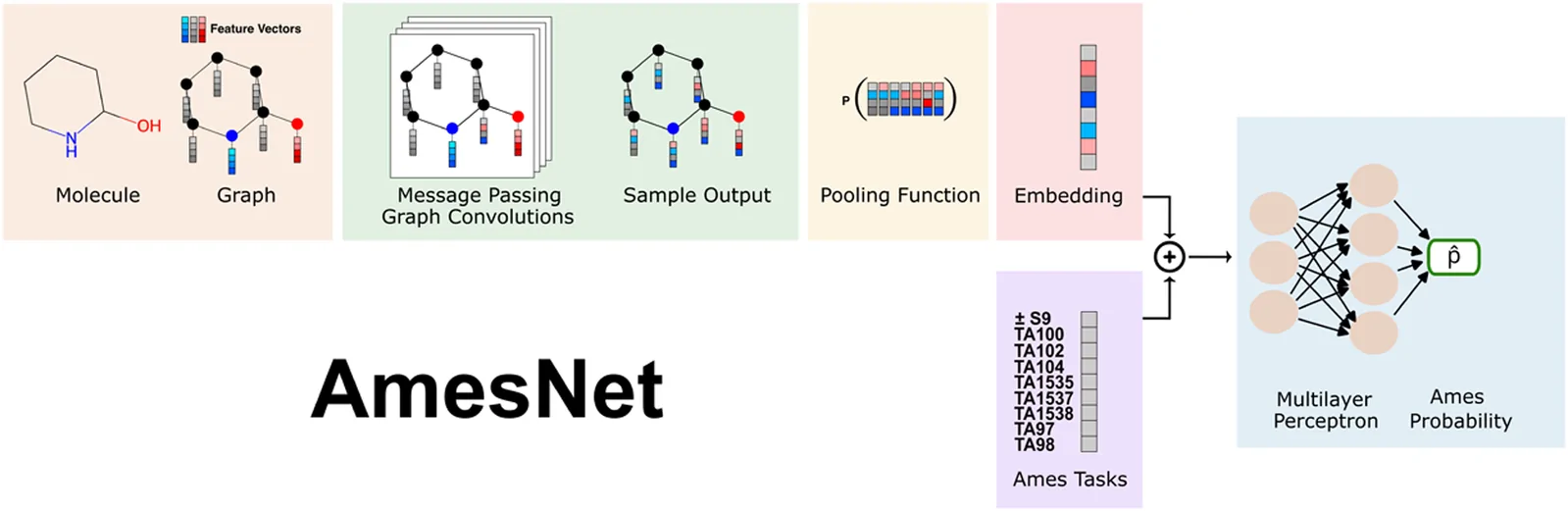

## Introduction

New small-molecule pharmaceuticals must undergo a suite of toxicity testing before entering clinical trials. The FDA and international regulators expect Ames genotoxicity data in regulatory submissions as described in the ICH S2­(R1) guideline.^1–3^ The Ames test under Good Laboratory Practice (GLP) conditions exceeds $10,000 per compound. This cost excludes the cost of chemical synthesis.^4^ Such high expenditure makes routine screening of large compound libraries impractical during early drug discovery. GLP Ames testing typically occurs near the regulatory submission stage after significant investment into the preclinical candidate.^5^ Preparing for human clinical trials takes approximately 3 years in the average small-molecule discovery timeline. This process can require >$10 million per candidate.^6^ An Ames test failure at this late stage results in years of wasted effort and millions of dollars in lost capital.

In response to such bottlenecks, the FDA and regulatory agencies around the world have formalized in silico approaches as a pathway to reduce traditional preclinical testing. The FDA Modernization Act now provides the legal framework for AI-based computer models to support and reduce wet-lab and animal testing for drug evaluation.^7^ Accordingly, the FDA established programs like the Artificial Intelligence Program for Toxicology (AI4TOX) to operationalize AI within safety reviews.^8^ Under this program the SafetAI initiative focuses on developing AI models for toxicological end points. DeepAmes was developed in this initiative to predict Ames mutagenicity for use within regulatory frameworks.^9,10^ Internationally, the ICH M7­(R2) guideline explicitly endorses two complementary (Q)­SAR methodologies for assessing Ames mutagenicity. These are an expert rule-based Structure–Activity Relationship (SAR) model and a statistical Quantitative Structure–Activity Relationship (QSAR) model trained on experimental Ames data. A negative prediction from both methodologies classifies an impurity as of no mutagenic concern and requires no further wet lab testing.^11^ The First and Second Ames/QSAR International Challenge Projects further propelled regulatory acceptance. During these projects, government agencies in the U.S., Japan, and Italy used standardized benchmarks in an attempt to improve model performance for Ames prediction.^12,13^

The Ames test uses specialized bacterial strains in growth-restrictive conditions to detect if a drug candidate might cause genetic mutations. Mutagenic potential is evidenced by bacterial growth after exposure to either the parent compound or its metabolic byproducts (Figure 1A).^1,2,14^ Effective screening therefore depends on high sensitivity to avoid misclassifying a dangerous molecule as safe. Meeting this requirement is a challenge for Ames QSAR models. Low sensitivity is the critical failure point of current models because it leads to more genotoxic compounds advancing undetected.^12,13^ Such failures are amplified when AI models encounter novel chemical structures distinct from their training data. This challenge in novel chemical space is known as out-of-domain (OOD) generalization. Achieving operational Ames model utility simultaneously requires high sensitivity and OOD generalization so that no genotoxic compound escapes detection when screening novel chemical libraries.^12,13^ Benchmark data from the Second Ames/QSAR International Challenge Projects revealed a significant collapse in sensitivity for current Ames models when encountering novel chemotypes.^13^ The project yielded a participant average sensitivity of only 0.46 on the OOD test set. High-profile models like the FDA’s DeepAmes achieved a sensitivity of 0.47 and MIT’s ChemProp yielded 0.32.^13^ Attempts to increase sensitivity have forced trade-offs in overall model performance, which can be measured by balanced accuracy (BA). DeepAmes more recently reports a sensitivity of 0.87 with a corresponding BA of 0.52, providing little predictive signal.^9,10^ These results demonstrate that current methods cannot maintain the sensitivity required for the reliable safety screening of novel drug candidates. Such unreliability creates a pathway for unidentified genotoxic molecules to advance into later stage drug development and potentially waste years and millions of dollars in resources.

**Figure 1:**
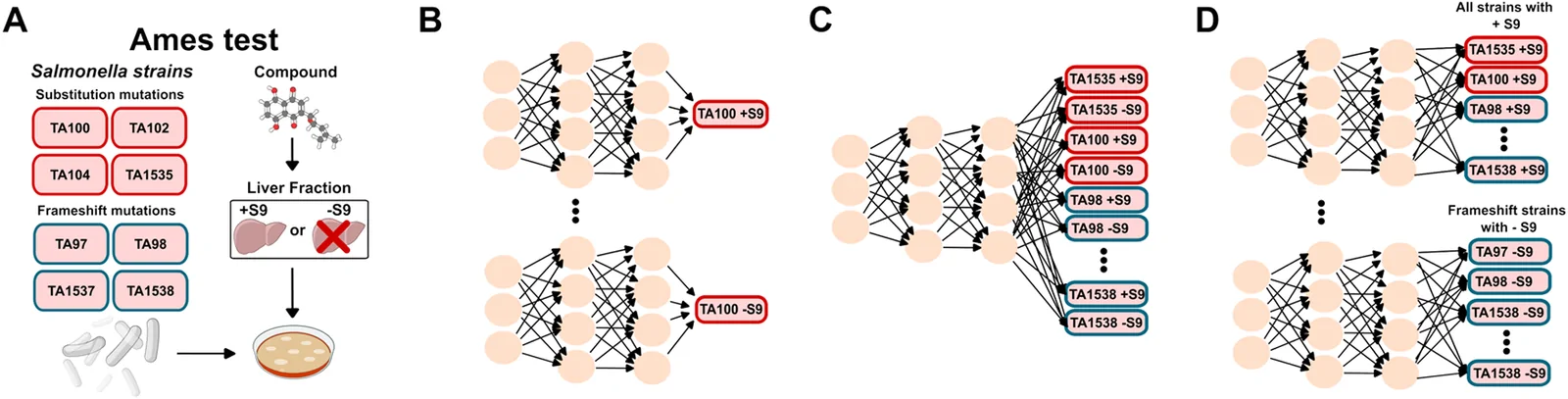
Ames test and predictive algorithms. (A) Diagram depicting the Ames test, where the rectangles represent eight strains commonly used. Compounds are tested in the presence or absence of a liver fraction (±S9), which mimics metabolic activation in the human body. (B) Single-task Ames QSAR algorithm (STL) trained to predict a strain-specific feature, with or without metabolic activation (±S9). (C) Ungrouped multitask Ames QSAR algorithm (uMTL), trained to predict all strain-specific features, with or without metabolic activation (±S9). (D) Grouped multitasked Ames QSAR algorithm (gMTL), trained to predict subsets such as substitution strains-specific features with or without metabolic activation (±S9).^19^

To our best knowledge, all existing AI models for Ames prediction fall under the archetype of Task-Unconditioned Modeling. In this archetype, predictions are generated without the use of an input conditioning channel for explicit assay states like bacterial strain or metabolic activation (±S9). The pervasive sensitivity gap across current Ames QSAR models necessitates a new modeling framework for maintaining robust performance in novel chemical space. Table 1 classifies the major Ames QSAR learning paradigms by functional form and distinguishes Task-Conditioned from Task-Unconditioned approaches.

**Table 1:** Learning Paradigms Classified by Functional Form and Task Conditioning

Model Type	Function	Classification
Task-Conditioned Learning (TCL)	*f*(molecule, task) → *P*	Task-Conditioned
Multi-Task Learning (MTL)	*f*(molecule) → [*P*_1_, ..., *P*_*N*_]	Task-Unconditioned
Single-Task Learning (STL)	*f*(molecule) → *P*	Task-Unconditioned

We introduce AmesNet, a Task-Conditioned Learning (TCL) model that concatenates one-hot strain and ±S9 descriptors with a molecular embedding. This conditional-learning approach is established in multitask learning. To our knowledge, it has not previously been applied to Ames QSAR. AmesNet uses an adaptation of ChemPrint from Model Medicines’ GALILEO platform as the molecular encoder (Figure 2). We selected ChemPrint because Ames benchmarking requires robust performance on structurally novel compounds, and ChemPrint was previously validated in OOD-rich discovery settings: on-target hit rates up to 100%, selectivity improvements exceeding 15,000-fold, and advancement of MDL-001 to preclinical proof-of-concept across several indications.^15–18^ AmesNet’s primary intended use is early stage triage in ultralarge virtual screening to flag mutagenic liabilities across large libraries before synthesis. AmesNet can also serve as the statistical QSAR model paired with an expert rule-based system under ICH M7­(R2) for late-stage regulatory assessment. We propose this framework to close the existing performance gap of Ames QSAR models and enable high-confidence mutagenicity screening in novel chemical space.

**Figure 2:**
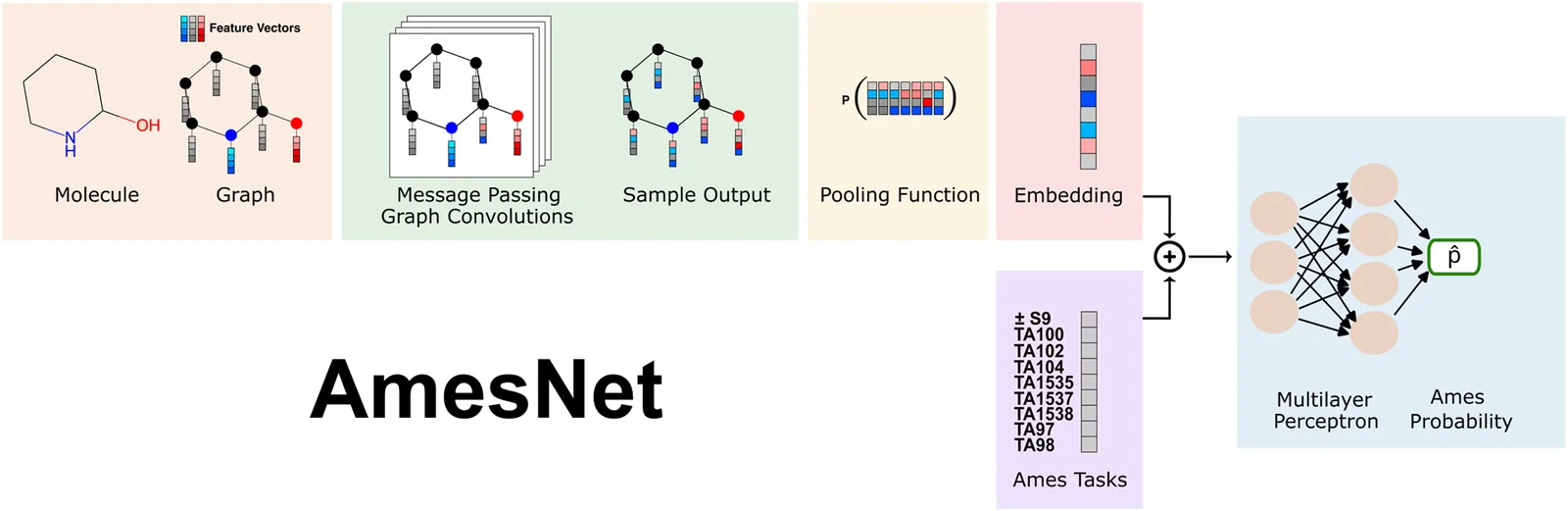
AmesNet TCL architecture. AmesNet is a dual branch architecture containing a graph convolutional neural network and an Ames task conditioning channel conditioned on strain and ±S9.

The choice of test data is critical for assessing the architectures defined in Table 1. We evaluate on two OOD test data sets. Our primary data set comes from Lui et al. and our secondary data set comes from Foil et al.^19,20^ Lui et al. is the superior data set because it retains strain and ± S9 metabolic-activation information. The Foil et al. data set reports only a single meta-level Ames classification. This single classification does not represent a real-world output because the Ames assay does not produce a single result per compound. Standard GLP clinical-readiness Ames assessment must evaluate a compound across 5 strains and 2 ±S9 conditions for a total of 10 strain-specific and metabolic-activation-specific results, a subset of the 16 (strain, ±S9) tasks evaluated in this work.^2,3^ The strain and S9 context that defines the mechanistic basis of an Ames result is therefore absent in Foil et al. Recent work from Dmitriev et al. discusses the importance of the mechanistic information from strain-level and metabolic-activation (±S9) labels that consensus or meta-level mutagenicity classification obscures.^21^ A meta-label assigned from a single assay combination may be incorrect because the full panel could produce a different result, and this potential error is difficult to verify without the underlying assay-level data. We therefore treat strain- and S9-resolved data from Lui et al. as the primary benchmark for mechanistically meaningful Ames prediction and present collapsed-label Foil et al. data as a secondary, lower-resolution test in the Supporting Information.

We execute a two-tiered benchmarking strategy to assess AmesNet’s overall performance and to isolate the contribution of TCL for Ames prediction. First, we measure AmesNet’s end-to-end performance relative to established learning frameworks: Single-Task Learning (STL), Ungrouped Multi-Task Learning (uMTL), and Grouped Multi-Task Learning (gMTL) (Figure 1B–D).^19^ In this benchmark, we include STL instantiations of ChemProp, GROVER, DeepAmes, a Multilayer Perceptron (MLP), and a Random Forest (RF).^9,10,22,23^ This comparison quantifies AmesNet’s overall performance advantage relative to these comparators. Second, we isolate the contribution of the TCL framework by augmenting established encoders with the conditioning channel: message-passing networks, graph transformers, and decision trees. In this benchmark, we include TCL instantiations of ChemProp, GROVER, and a RF. This comparison quantifies the performance gains attributable to TCL. We assess performance for each benchmark through the concurrent evaluation of generalization and classification metrics on withheld OOD test sets. Sensitivity is reported as the primary metric. Additionally, we utilize BA and Matthews correlation coefficient (MCC) to verify the model’s overall predictive robustness. Across all benchmarks, AmesNet demonstrates substantial improvements in sensitivity, BA, MCC, and OOD generalization, outperforming both the Task-Unconditioned Models in the first benchmark and the encoder-swaps in the second benchmark. These results demonstrate that AmesNet and its TCL framework offer enhanced predictive performance in novel chemical space over alternatives.

The performance of AmesNet substantially addresses the OOD sensitivity gap that has limited the utility of Ames QSAR models. AmesNet provides a more reliable pathway for higher-confidence safety screening at the earliest stages of discovery. This framework offers a notably improved alternative to current in silico approaches and serves to mitigate the risk of late-stage failure due to undetected genotoxicity. This TCL paradigm supports higher-confidence screening of massive chemical libraries and has the potential to transform a late-stage safety liability into earlier-stage advantage.

## Methods

### Data

#### Data Set Source: Lui et al.

The data set was compiled by Lui et al. from four individual data sources: ISSSTY, OASIS, EFSAP, and MHLW.^19^ The data set included Ames test annotations from eight Salmonella strains, each corresponding to a specific mutation type. The base-pair substitution strains are TA100, TA102, TA104, and TA1535. The frameshift mutation strains are TA1537, TA1538, TA97, and TA98. Assays were conducted in the presence or absence of S9 fraction.

#### Data Set Source: Foil et al.

The data set was compiled by Foil et al. from 15 individual public genotoxicity data sources containing Ames test data without strain/ ± S9-level information: AMINES, AmesFormer, Benfenati, BfR, CAESAR, ECVAMneg, ECVAMpos, EFSA_PestGentox, Hansen, ISS, ISSSTY, Karamertzanis, OCHEM, SARPY, and Xu.^20^ Substance-level hit calls were aggregated conservatively: any positive result was called positive and only unanimous negatives were called negative.

#### Data Set Cleaning: Lui et al.

Lui et al. created the data set by dropping rows whose compounds do not contain carbon atoms and rows whose compounds contain any metals or metalloids, allowing only H, C, N, O, F, P, S, Cl, Br, and I. Lui et al. provided these compounds in SDF file format. The molecules in the data set were converted from SDF to SMILES format. They were subsequently standardized with respect to salts, charge, stereochemistry, and tautomerism. Standardization and validation were performed using a proprietary in-house pipeline whose implementation is not disclosed. SMILES strings containing multiple fragments were desalted by retaining only the single largest organic fragment by heavy-atom count. Charge was set by canonicalizing protonation states so the strongest acidic sites were ionized first. All tautomers were canonicalized, covering keto–enol, enamine-imine, amide-imidic acid (lactam-lactim), hydroxypyridine-pyridone and related ring–chain heterocyclic shifts, nitroso-oxime, nitro/aci-nitro, azo-hydrazone, thione-thiol, phosphonate and sulfonate proton shifts, and 1,3- and 1,5-proton migrations across conjugated systems. SMILES were enumerated, scored, and collapsed to one canonical form. A placeholder column was created and populated with the canonical nonisomeric SMILES form. Stereochemistry was removed only for this column. Next, invalid structures were removed. All (compound, strain, S9) triplets with conflicting Ames end points were removed from the data set. Duplicate triplets sharing identical end points were collapsed to a single instance. Compounds were filtered by molecular weight in the range [100, 1000] Da to retain only small molecules. The data set was prepared for model training, validation, and testing according to the predefined OOD data splits in the comparator publication.^19^ Data leakage from the Training/Validation data into the Test data was eliminated by dropping Test compounds whose canonical nonisomeric SMILES exactly matched any in the Training/Validation data. These filtering steps reduced the training/validation set from 43,897 to 40,129 data points and the test set from 4,838 to 4,208 data points.

#### Data Set Cleaning: Foil et al.

Foil et al. provided the data as an Excel file with SMILES. This data set differs from the Lui et al. data in that it lacks S9 and strain-level information. We filtered the data to retain only rows with an associated Ames test readout. The SMILES were subsequently standardized with respect to salts, charge, stereochemistry, and tautomerism. Standardization and validation were performed using a proprietary in-house pipeline whose implementation is not disclosed. SMILES strings containing multiple fragments were desalted by retaining only the single largest organic fragment by heavy-atom count. Charge was set by canonicalizing protonation states so the strongest acidic sites were ionized first. All tautomers were canonicalized, covering keto–enol, enamine-imine, amide-imidic acid (lactam-lactim), hydroxypyridine-pyridone and related ring–chain heterocyclic shifts, nitroso-oxime, nitro/aci-nitro, azo-hydrazone, thione-thiol, phosphonate and sulfonate proton shifts, and 1,3- and 1,5-proton migrations across conjugated systems. SMILES were enumerated, scored, and collapsed to one canonical form. A placeholder column was created and populated with the canonical nonisomeric SMILES form. Stereochemistry was removed only for this column. Next, invalid structures were removed. Exact duplicates were then dropped on the canonical nonisomeric SMILES column, retaining one record per structure. Ames label conflicts were resolved by assigning positive if at least one record was positive and negative if all records were negative. Compounds lacking a carbon atom were dropped. Compounds containing any metals or metalloids were also dropped, restricting the allowed element set to H, C, N, O, F, P, S, Cl, Br, and I. Compounds were then filtered by molecular weight to the range [100, 1000] Da to retain only small molecules. Data leakage from the Lui et al. training/validation data into the Foil et al. test data was eliminated. Any Foil et al. test compound whose canonical nonisomeric SMILES exactly matched a Lui et al. training/validation entry was dropped. These filtering steps reduced the test set from 22,627 to 8,717 data points.

#### Data Set EDA: Lui et al. and Foil et al.

The exploratory analysis characterized how mutagenicity labels are distributed. We counted compounds by outcome class to assess class composition at the whole-data set level. For the Lui data sets, we extended this analysis into a stratified view by bacterial strain and metabolic activation condition. These counts are available as clean summary tables in the Supporting Information.

We performed applicability domain (AD) analysis using Lui et al. (Train/Val) as the reference set and both Lui et al. (Test) and Foil et al. (Test) as the AD sets. We generated ECFP4 2048 fingerprints from SMILES. For each test molecule, we computed the maximum Tanimoto similarity to the Lui-Train/Val fingerprint pool. We summarized each test set’s similarity distribution with mean, median, and standard deviation. We then calculated the proportion of molecules outside the applicability domain at fixed similarity cutoffs (0.50, 0.60, and 0.65). This summary table is available in the Supporting Information.

#### Data Set Featurization: Lui et al. and Foil et al.

Each compound is represented as a geometric molecular graph. Nodes correspond to atoms, and edges correspond to chemical bonds. This representation captures the compound’s structural information. Atomic features are encoded as one-hot vectors and provided as node attributes.

ECFP4 fingerprints were used only for applicability-domain analysis and were not used as inputs to AmesNet. For AmesNet, standardized SMILES were converted to atom-bond molecular graphs using a proprietary in-house graph-construction pipeline. The ChemPrint encoder applies graph convolutions over these graphs rather than over circular fingerprints.

Nine one-hot encoded context features represent the Ames assay conditions and are provided through the state conditioning channel. The experimental conditions consist of the Salmonella strain and the presence or absence of S9 metabolic activation. Each data point corresponds to a (compound, strain, S9) triplet with a binary ground-truth Ames label (±).

### AmesNet Architecture

#### Task-Conditioned Dual Branch

AmesNet consists of two input branches. The first branch encodes molecular structure using an atomic-level graph encoder that produces a 512-dimensional molecular embedding. The second branch encodes nine one-hot Ames assay context features specifying the Salmonella strain and the presence or absence of S9 metabolic activation. The molecular embedding and context feature vectors are concatenated and passed to a multilayer perceptron for prediction. The molecular branch captures structural information, while the context branch enables conditional mutagenicity prediction. All AmesNet parameters are learned end-to-end from random initialization, and no weights are imported or frozen.

### Model Development Operations

#### Hyperparameter Optimization

Grid search was used to optimize hyperparameters during model training and validation.

#### Compute Resources

All model training and inference were performed on an NVIDIA A100 40GB GPU.

### Reference Models

#### Task-Unconditioned Models

AmesNet was benchmarked against three Task-Unconditioned Ames QSAR frameworks: single-task learning (STL), ungrouped multitask learning (uMTL), and grouped multitask learning (gMTL). The multilayer perceptron (MLP) architectures for STL, uMTL, and gMTL follow the reference implementation.^19^ In addition, four architectures (DeepAmes, ChemProp, GROVER, and a Random Forest) were evaluated as STL baselines by training 16 task-specific models per architecture (bacterial strain x ±S9).^9,10,22,23^ Each model was trained end-to-end.

#### Task-Conditioned Encoder-Swap Models

AmesNet was also benchmarked by injecting the task conditioning channel into a Random Forest, ChemProp, and GROVER_large_.^22,23^ Each encoder-swap model was trained end-to-end.

### Metrics and Bootstrapping

#### Lui et al. Test Set

Multitask binary classification performance was evaluated across 16 Ames tasks. Each task was defined as a unique combination of bacterial strain and ±S9. Sensitivity, BA, and Matthews correlation coefficient (MCC) were computed per task and are the principal metrics reported in the main text. Specificity and PPV were also computed per task and are reported in the Supporting Information. Each metric was aggregated using a sample-size weighted average, with weights proportional to the number of observations per task. Uncertainty was estimated with a within-task stratified bootstrap (*n* = 1,000). Within each task, positives and negatives were resampled with replacement to match the original positive and negative counts of that task’s test set, preserving the empirical class ratio and total sample size in every replicate. Metrics were recomputed per task and aggregated using the same weights to yield one bootstrap replicate of the estimator. The 95% confidence interval was defined by the 2.5th and 97.5th percentiles of the bootstrap distribution.

#### Foil et al. Test Set

Binary classification performance was evaluated per model on the Foil end point. For each model, duplicate SMILES in the model’s binary prediction output were collapsed by taking the maximum binary prediction per SMILES before metric computation. Sensitivity, BA, MCC, Specificity and PPV were computed and are reported in the Supporting Information. Uncertainty was estimated with a stratified bootstrap (*n* = 1,000). Positives and negatives were resampled with replacement to match the original positive and negative counts of the model’s data set, preserving the empirical class ratio and total sample size in every replicate. Metrics were recomputed on each bootstrap replicate. The 95% confidence interval was defined by the 2.5th and 97.5th percentiles of the bootstrap distribution.

## Results

### Benchmark 1.1 (Lui et al.): AmesNet vs Task-Unconditioned Modeling Paradigms

#### Benchmark 1.1: Sensitivity on Lui et al. Test Set

Benchmark 1.1 evaluated the performance of AmesNet against established Task-Unconditioned Ames QSAR frameworks (STL, uMTL, and gMTL) on sensitivity in an OOD setting. OOD sensitivity and 95% confidence intervals were estimated as described in Methods (Figure 3A). AmesNet achieved a sensitivity of 0.72 (95% CI: 0.68–0.76), the highest reported among all models evaluated in Benchmark 1.1. Task-Unconditioned Models showed consistently lower sensitivity. Within the STL framework, the STL-MLP achieved 0.48 (95% CI: 0.43–0.53), STL-ChemProp achieved 0.54 (95% CI: 0.49–0.59), STL-GROVER achieved 0.54 (95% CI: 0.49–0.59), STL-DeepAmes achieved 0.69 (95% CI: 0.64–0.73), and STL-RF achieved 0.40 (95% CI: 0.36–0.44). Within the uMTL framework, the uMTL-MLP achieved 0.56 (95% CI: 0.51–0.62). Within the gMTL framework, the gMTL-MLP achieved 0.56 (95% CI: 0.52–0.61). AmesNet achieved sensitivity improvements of +0.32 (80%) over STL-RF, +0.24 (50%) over STL-MLP, +0.18 (33%) over STL-ChemProp, +0.18 (33%) over STL-GROVER, +0.03 (4%) over STL-DeepAmes, +0.16 (29%) over uMTL-MLP, and +0.16 (29%) over gMTL-MLP. AmesNet exhibits nonoverlapping 95% confidence intervals across sensitivity measurements relative to STL-GROVER, STL-ChemProp, STL-RF, STL-MLP, uMTL-MLP, and gMTL-MLP baselines.

**Figure 3:**
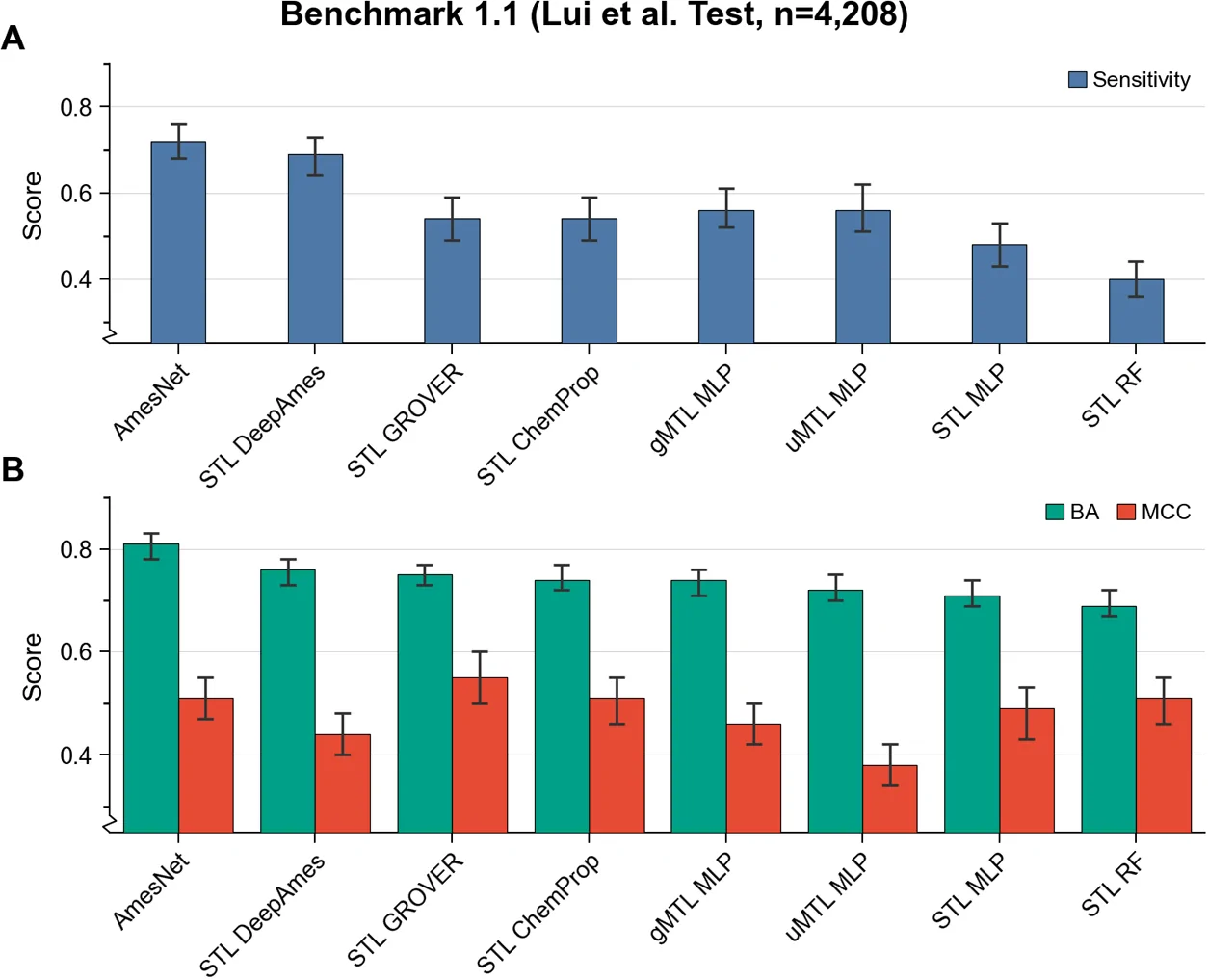
Sample-size-weighted metrics on the fixed Lui et al. test set. (A) Sensitivity. (B) Balanced accuracy (BA) and Matthews correlation coefficient (MCC). Error bars indicate 95% confidence intervals from a within-task stratified bootstrap (*n* = 1,000) across the 16 Ames tasks. The *y*-axis is rescaled; the full metric range is 0–1.

#### Benchmark 1.1: Balanced Accuracy on Lui et al. Test Set

OOD balanced accuracy was calculated with 95% confidence intervals (Figure 3B). AmesNet achieved a balanced accuracy of 0.81 (95% CI: 0.78–0.83). AmesNet demonstrates a substantial improvement in balanced accuracy over all models, as evidenced by nonoverlapping 95% bootstrap confidence intervals in all but one model. Within the STL framework, the STL-RF achieved 0.69 (95% CI: 0.67–0.72), STL-MLP achieved 0.71 (95% CI: 0.69–0.74), STL-ChemProp achieved 0.74 (95% CI: 0.72–0.77), STL-GROVER achieved 0.75 (95% CI: 0.73–0.77), and STL-DeepAmes achieved 0.76 (95% CI: 0.73–0.78). Within the uMTL framework, the uMTL-MLP achieved 0.72 (95% CI: 0.70–0.75). Within the gMTL framework, the gMTL-MLP achieved 0.74 (95% CI: 0.71–0.76). AmesNet achieved balanced accuracy improvements of +0.12 (17%) over STL-RF, +0.10 (14%) over STL-MLP, +0.07 (9%) over STL-ChemProp, +0.06 (8%) over STL-GROVER, +0.05 (7%) over STL-DeepAmes, +0.09 (13%) over uMTL-MLP, and +0.07 (9%) over gMTL-MLP.

#### Benchmark 1.1: Matthews Correlation Coefficient on Lui et al. Test Set

OOD Matthews Correlation Coefficient (MCC) was calculated with 95% confidence intervals (Figure 3B). AmesNet achieved a MCC of 0.51 (95% CI: 0.47–0.55). Within the STL framework, the STL-RF achieved 0.51 (95% CI: 0.46–0.55), STL-MLP achieved 0.49 (95% CI: 0.43–0.53), STL-ChemProp achieved 0.51 (95% CI: 0.46–0.55), STL-GROVER achieved 0.55 (95% CI: 0.50–0.60), and STL-DeepAmes achieved 0.44 (95% CI: 0.40–0.48). Within the uMTL framework, the uMTL-MLP achieved 0.38 (95% CI: 0.34–0.42). Within the gMTL framework, the gMTL-MLP achieved 0.46 (95% CI: 0.42–0.50). AmesNet achieves higher MCC than four models, equivalent MCC to two models, and noninferior MCC to one model, as evidenced by nonoverlapping 95% bootstrap confidence intervals. AmesNet achieved MCC changes of +0.07 (16%) over STL-DeepAmes, +0.02 (4%) over STL-MLP, +0.00 (0%) over STL-RF, +0.00 (0%) over STL-ChemProp, −0.04 (−7%) over STL-GROVER, +0.13 (34%) over uMTL-MLP, and +0.05 (11%) over gMTL-MLP.

#### Benchmark 1.1: Structural Enrichment of True-Positive Predictions in Lui et al. Test Set

Figure 4 summarizes mutagenic substructures present in compounds that AmesNet classified as true positives but that Task-Unconditioned Models misclassified as false negatives. The largest counts were observed for planar aromatic intercalators (*n* = 27–51), aromatic amines (*n* = 22–37), and aromatic *N*-heterocycles (*n* = 17–28). Smaller but recurrent categories included epoxides (*n* = 5–23), α,β-unsaturated carbonyls (*n* = 5–14), nitro-aromatics (*n* = 4–10), aromatic ring *N*-oxides (*n* = 4–10), azo groups (*n* = 4–6), aliphatic halogens (*n* = 2–6), 1,2-dihydroxy aromatic rings (*n* = 2–7), polyfunctional alkylators (*n* = 3–5), aryl halides (*n* = 0–3), and nitroso groups (*n* = 1–2). These patterns show that false negatives in Task-Unconditioned Models are concentrated in a small set of well-known mutagenic alert classes and that AmesNet correctly identifies many of these cases.

**Figure 4:**
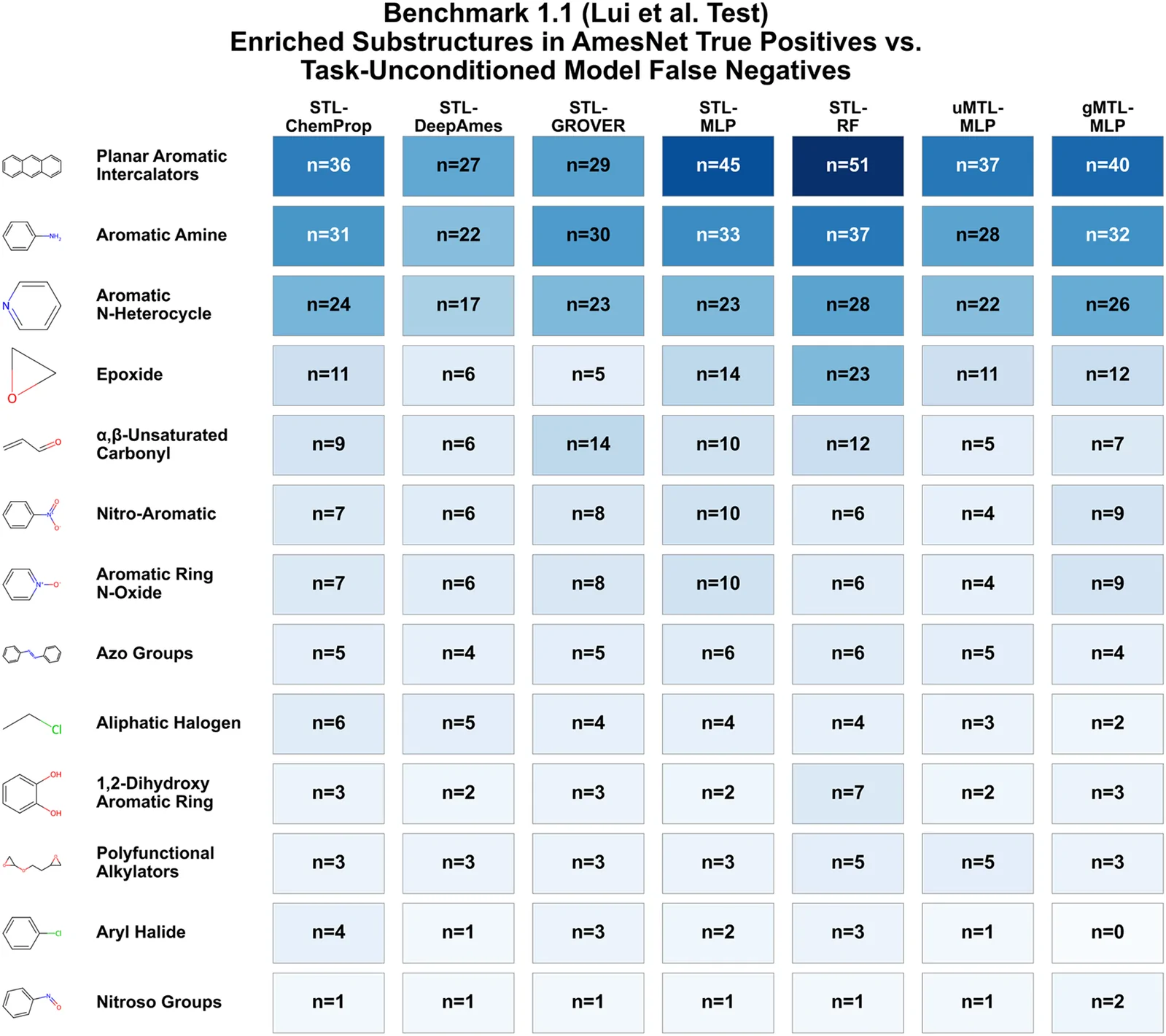
Enriched Substructures in AmesNet True Positives vs Task-Unconditioned Model False Negatives. Mutagenic substructures observed in compounds correctly predicted by AmesNet but misclassified by unconditioned baseline models (gMTL-MLP, uMTL-MLP, STL-MLP, STL-RF, STL-ChemProp, STL-DeepAmes, STL-GROVER). Values (*n*) indicate the number of unique substructure-containing compounds where AmesNet achieved true positives while comparison models achieved false negatives. Color intensity encodes *n*, with darker shades indicating a greater number of substructure-containing compounds.

### Benchmark 1.2 (Lui et al.): Encoder-Swaps within TCL

#### Benchmark 1.2: Sensitivity on Lui et al. Test Set

Benchmark 1.2 isolated the contribution of the TCL framework by augmenting three established encoders with the conditioning channel to create TCL-ChemProp, TCL-GROVER, and TCL-RF. AmesNet achieved the highest sensitivity on the OOD test set (Figure 5A), reaching a sensitivity of 0.72 (95% CI: 0.68–0.76). TCL-ChemProp improved sensitivity relative to STL-ChemProp, increasing from 0.54 (95% CI: 0.49–0.59) to 0.64 (95% CI: 0.59–0.68), a +0.10 absolute gain (19% relative). TCL-RF improved sensitivity relative to STL-RF, increasing from 0.40 (95% CI: 0.36–0.44) to 0.50 (95% CI: 0.45–0.55), a +0.10 absolute gain (25% relative). By contrast, TCL-GROVER showed no material sensitivity change relative to STL-GROVER (0.54, 95% CI: 0.49–0.59 vs 0.55, 95% CI: 0.50–0.58). Together, these results indicate that within the TCL paradigm, AmesNet’s molecular representation provides a sensitivity advantage over leading message-passing and graph transformer alternatives. The TCL framework also confers a material sensitivity lift for TCL-ChemProp relative to STL-ChemProp and TCL-RF relative to STL-RF.

**Figure 5:**
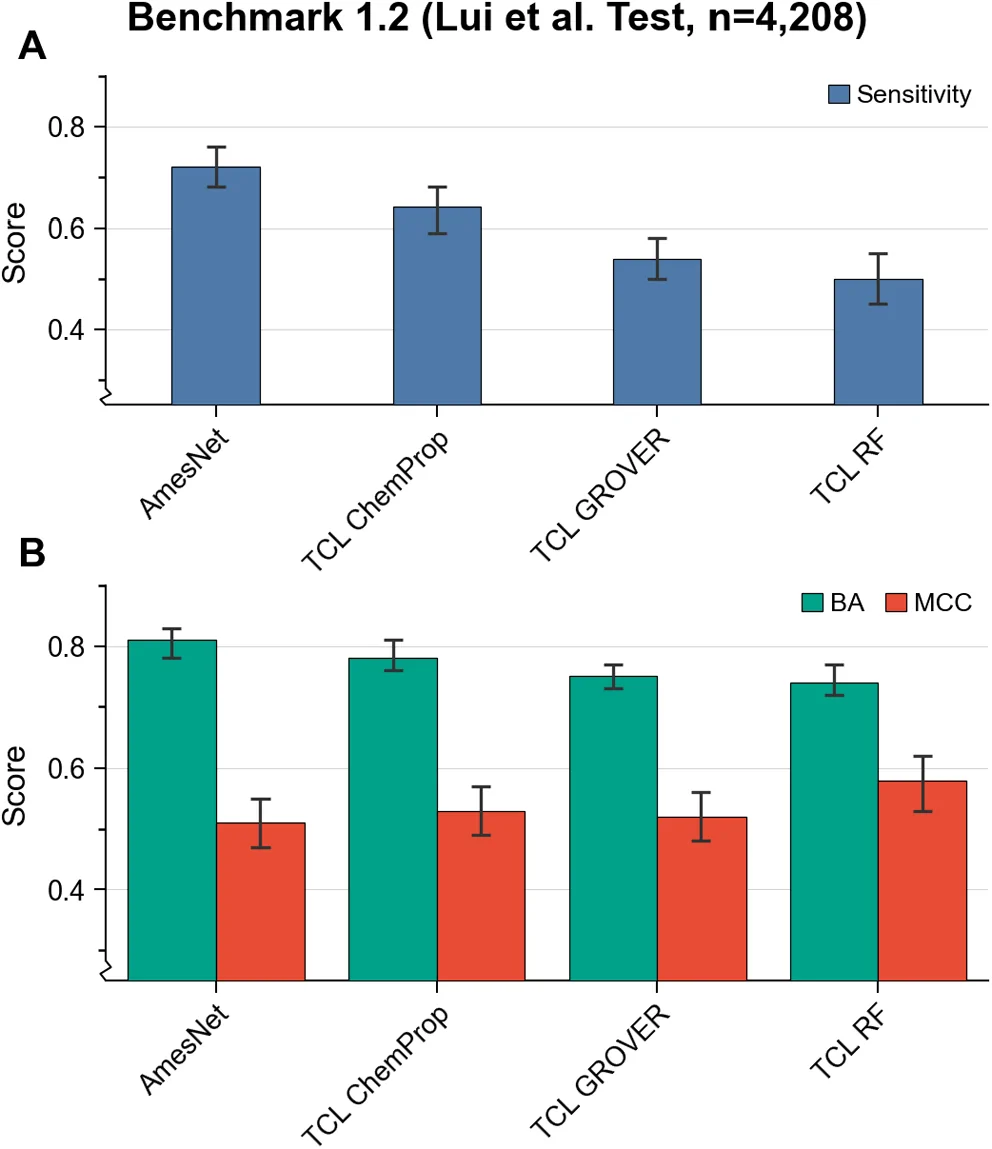
Sample-size-weighted metrics on the fixed Lui et al. test set. (A) Sensitivity. (B) Balanced accuracy (BA) and Matthews correlation coefficient (MCC). Error bars indicate 95% confidence intervals from a within-task stratified bootstrap (*n* = 1,000) across the 16 Ames tasks. The *y*-axis is rescaled; the full metric range is 0–1.

#### Benchmark 1.2: Balanced Accuracy on Lui et al. Test Set

Balanced accuracy on the OOD test set was calculated and 95% confidence intervals were reported (Figure 5B). AmesNet achieved a balanced accuracy of 0.81 (95% CI: 0.78–0.83). STL-ChemProp achieved 0.74 (95% CI: 0.72–0.77) and TCL-ChemProp achieved 0.78 (95% CI: 0.76–0.81), a +0.04 absolute gain (5% relative). STL-RF achieved 0.69 (95% CI: 0.67–0.72) and TCL-RF achieved 0.74 (95% CI: 0.72–0.77), a +0.05 absolute gain (7% relative). TCL-GROVER showed no change in balanced accuracy compared to STL-GROVER with 0.75 (95% CI: 0.73–0.77) and 0.75 (95% CI: 0.73–0.77), respectively. Together, these results show that AmesNet achieves the strongest balanced accuracy and that the TCL framework confers a material balanced accuracy lift for TCL-ChemProp relative to STL-ChemProp and TCL-RF relative to STL-RF.

#### Benchmark 1.2: Matthews Correlation Coefficient on Lui et al. Test Set

MCC on the OOD test set was calculated and 95% confidence intervals were reported (Figure 5B). AmesNet achieved a MCC of 0.51 (95% CI: 0.47–0.55). STL-ChemProp achieved 0.51 (95% CI: 0.46–0.55) and TCL-ChemProp achieved 0.53 (95% CI: 0.49–0.57), a +0.02 absolute gain (4% relative). STL-RF achieved 0.51 (95% CI: 0.46–0.55) and TCL-RF achieved 0.58 (95% CI: 0.53–0.62), a +0.07 absolute gain (14% relative). STL-GROVER achieved 0.55 (95% CI: 0.50–0.60) and TCL-GROVER achieved 0.52 (95% CI: 0.48–0.56). Together, these results show that the TCL framework confers a MCC lift for TCL-ChemProp relative to STL-ChemProp and TCL-RF relative to STL-RF.

#### Benchmark 1.2: Structural Enrichment of True-Positive Predictions in Lui et al. Test Set

Structural enrichment analysis (Figure 6) highlights mutagenic substructures that AmesNet recovers as true positives but that TCL-ChemProp, TCL-GROVER, and TCL-RF miss as false negatives. The most frequently missed classes were planar aromatic intercalators (*n* = 24–40), aromatic amines (*n* = 24–37), followed by aromatic N-heterocycles (*n* = 16–26). Additional recurrent alerts included epoxides (*n* = 1–13), α,β-unsaturated carbonyls (*n* = 6–9), nitro-aromatics and aromatic ring *N*-oxides (*n* = 3–4 each), azo groups (*n* = 5–9), aliphatic halogens (*n* = 4–5), 1,2-dihydroxy aromatic rings (*n* = 3–7), polyfunctional alkylators (*n* = 0–4), aryl halides (*n* = 2–4), and nitroso groups (*n* = 0–2). Relative to STL-ChemProp, TCL-ChemProp shows broad recovery for planar aromatic intercalators (36 to 25), aromatic amines (31 to 24), aromatic *N*-heterocycles (24 to 16), epoxides (11 to 1), α,β-unsaturated carbonyls (9 to 6), nitro-aromatics (7 to 3), aromatic ring *N*-oxides (7 to 3), azo groups (5 to 5), aliphatic halogens (6 to 5), 1,2-dihydroxy aromatic rings (3 to 3), polyfunctional alkylators (3 to 0), aryl halides (4 to 2), and nitroso groups (1 to 0). Relative to STL-RF, TCL-RF shows broad recovery for planar aromatic intercalators (51 to 40), aromatic amines (37 to 37), aromatic *N*-heterocycles (28 to 26), epoxides (23 to 13), α,β-unsaturated carbonyls (12 to 9), nitro-aromatics (6 to 4), aromatic ring N-oxides (6 to 4), azo groups (6 to 9), aliphatic halogens (4 to 4), 1,2-dihydroxy aromatic rings (7 to 7), polyfunctional alkylators (5 to 4), aryl halides (3 to 4), and nitroso groups (1 to 1). Recovery from STL-GROVER to TCL-GROVER shows broad recovery for planar aromatic intercalators (29 to 24), aromatic amines (30 to 26), aromatic *N*-heterocycles (23 to 16), epoxides (5 to 4), α,β-unsaturated carbonyls (14 to 9), nitro-aromatics (8 to 4), aromatic ring *N*-oxides (8 to 4), azo groups (5 to 7), aliphatic halogens (4 to 4), 1,2-dihydroxy aromatic rings (3 to 6), polyfunctional alkylators (3 to 2), aryl halides (3 to 3), and nitroso groups (1 to 2). Together, these patterns indicate that within the TCL paradigm, AmesNet’s molecular representation more reliably recognizes mutagenic alert classes than TCL-ChemProp, TCL-GROVER, and TCL-RF. These results also show that the TCL framework can reduce conserved false-negative failures in established encoders.

**Figure 6:**
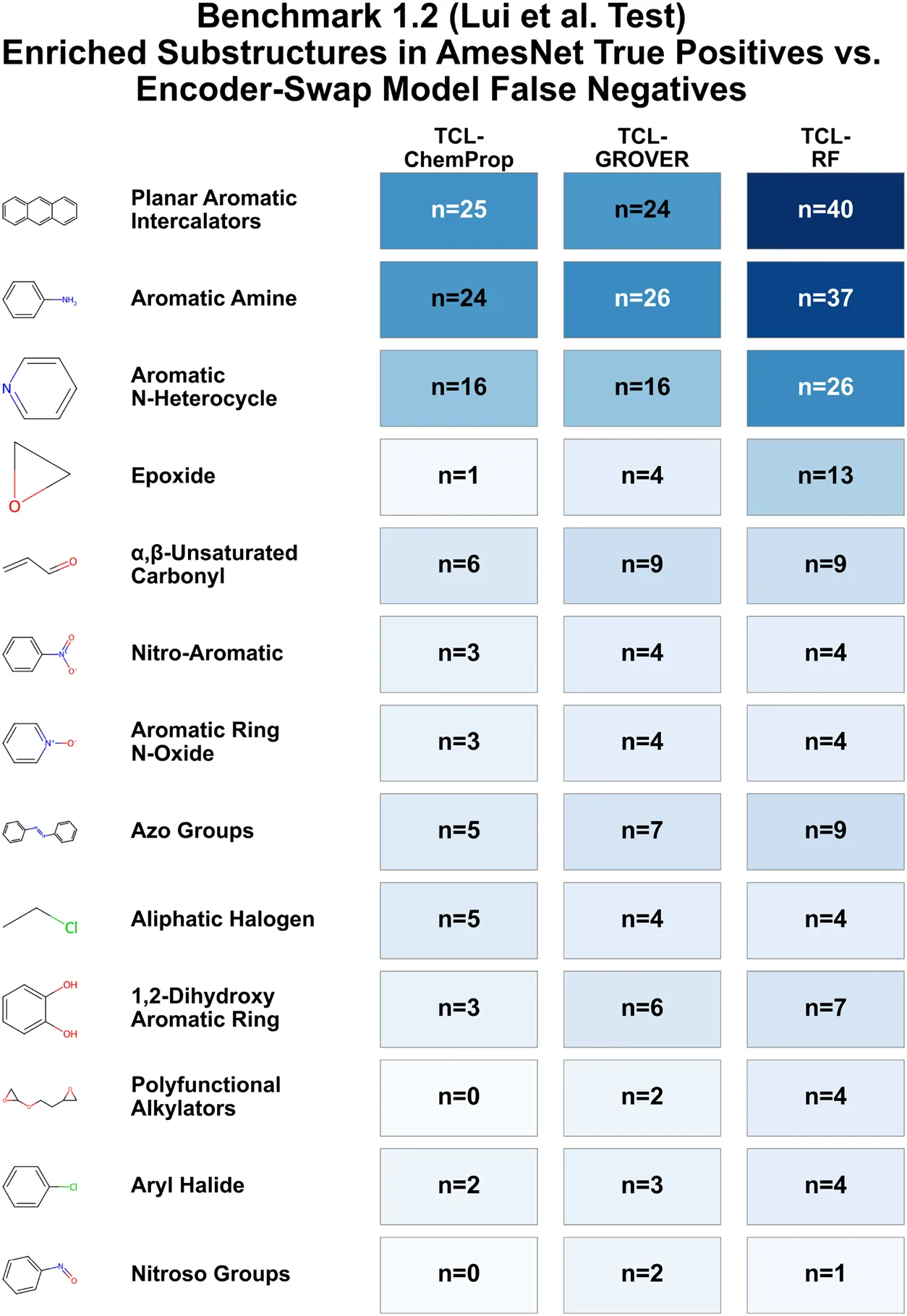
Enriched Substructures in AmesNet True Positives vs Encoder-Swap Model False Negatives. Mutagenic substructures observed in compounds correctly predicted by AmesNet but misclassified by encoder-swap TCL models (TCL-ChemProp, TCL-GROVER, TCL-RF). Values (*n*) indicate the number of unique substructure-containing compounds where AmesNet achieved true positives while comparison models achieved false negatives. Color intensity encodes *n*, with darker shades indicating a greater number of substructure-containing compounds.

## Discussion

### Addressing the Performance Gap

#### OOD Sensitivity is a Failure Mode in Ames QSAR

The First and Second Ames/QSAR International Challenge Projects established a consistent failure mode for Ames QSAR models under OOD evaluation. Sensitivity fails to hold under chemical novelty assessment as demonstrated by the participant average sensitivity of 0.46. ChemProp and DeepAmes yielded sensitivities of 0.32 and 0.47 in that competition setting. Those competition results show that existing models do not deliver the sensitivity required for reliable safety screening in chemically novel space.

#### Benchmark 1.1 (Lui et al.): AmesNet Performance vs Task-Unconditioned Modeling

We target the sensitivity failure mode directly using an OOD benchmark. AmesNet shows that sensitivity drop-off is not inevitable under chemical novelty. Instead, this pattern reflects the limitations of the Task-Unconditioned Modeling paradigm that has defined all prior approaches. AmesNet achieves a sensitivity of 0.72 (95% CI: 0.68–0.76), a BA of 0.81 (95% CI: 0.78–0.83), and a MCC of 0.51 (95% CI: 0.47–0.55) (Figure 3). AmesNet ranks first for sensitivity and BA. Importantly, the sensitivity improvement of AmesNet is not achieved through a trade-off in overall discrimination. BA and MCC were used to assess trade-offs. AmesNet’s performance advantage over DeepAmes becomes clear once BA and MCC are also assessed. AmesNet achieves substantial sensitivity gains without sacrificing overall predictive robustness.

#### Benchmark 1.1 (Lui et al.): Chemistry of AmesNet False-Negative Recovery

Each compound the Task-Unconditioned Models miss as a false negative is a drug candidate lost to years of delay and millions in cost. We aim to identify the mutagenic substructures that drive these misses and that AmesNet alone recovers as true positives.

AmesNet’s true positives are concentrated in well-established mutagenic alert classes that are documented sources of false negatives in Ames QSAR modeling.^24,25^ The Lui et al. test set contains 560 Ames true positives (Supporting Information). Across 13 mutagenic substructure classes, AmesNet recovered 106 more true positives than DeepAmes. DeepAmes was the next-best model after AmesNet.

The largest single-class recoveries are observed for planar aromatic intercalators and aromatic amines (Figure 4). Planar aromatic intercalators and many aromatic *N*-heterocycles often drive mutagenicity through DNA intercalation and are associated with a frameshift mutation signature.^26^ These chemotypes are detected in the Ames frameshift-sensitive bacterial strains (e.g., TA1537, TA1538, TA97, and TA98) rather than in base-substitution strains. AmesNet’s identification of these motifs as true positives is consistent with strain-dependent signal capture that comparator models miss on this test set.

Similar context dependence arises for +S9 metabolic activation. Aromatic amines require +S9 metabolic activation to express mutagenicity.^27^ AmesNet’s identification of aromatic amines as true positives is consistent with classification that is responsive to the S9 activation assay context. One plausible interpretation is that AmesNet learns different decision rules across assay states rather than averaging them into a single unconditional boundary.

#### Benchmark 1.2 (Lui et al.): Encoder-Swaps within TCL

The contribution of the TCL framework to the overall success of AmesNet was isolated by injecting the same assay-conditioning channel (strain and ±S9) into three well-established molecular encoders to form TCL-ChemProp, TCL-GROVER, and TCL-RF. Similarly to AmesNet’s molecular encoder, ChemProp is classified as a message-passing network encoder. GROVER is classified as a graph transformer architecture and TCL-RF is classified as a decision tree. Performance of TCL-ChemProp, TCL-GROVER, and TCL-RF variants was compared to their STL counterparts to attribute the lift to the TCL framework (Figure 5).

TCL-ChemProp exhibits a clear sensitivity lift over STL-ChemProp with a 19% relative gain. TCL-RF also improved sensitivity relative to STL-RF with a 25% relative gain. This sensitivity lift is accomplished without a trade-off in overall performance, as supported by increases in both BA and MCC for TCL variants of ChemProp and RF. BA and MCC from STL-ChemProp to TCL-ChemProp show a 5% relative gain and a 4% relative gain, respectively. BA and MCC from STL-RF to TCL-RF show a 7% relative gain and a 14% relative gain, respectively.

These results show that the TCL framework confers a sensitivity, BA, and MCC lift for TCL variants of ChemProp and RF. However, TCL-GROVER shows no material change in performance relative to STL-GROVER across sensitivity, BA, and MCC. This suggests that the concatenated conditioning channel may couple more effectively with message-passing network embeddings than with transformer-derived embeddings. Another plausible explanation is that GROVER’s pretrained features are less responsive to explicit metadata concatenation. Even with the performance lift from the TCL framework, no model was as performant as AmesNet in both sensitivity and BA. This indicates that the success of AmesNet comes both from its TCL framework and its molecular encoder. Together, these results support TCL as a practical mechanism for improving OOD mutagen recovery without trade-off in overall classification robustness.

#### Benchmark 1.2 (Lui et al.): Chemistry of TCL False-Negative Recovery

The TCL framework earns its place only if it recovers true-positive Ames compounds that its Task-Unconditioned model counterpart missed as false negatives. Each additional Ames positive recovered is a drug candidate spared from years of delay and millions in cost. TCL-ChemProp, TCL-GROVER, and TCL-RF reduced false negatives relative to their STL counterparts. AmesNet remained the strongest model overall.

We computed each TCL gain by subtracting the Figure 6 counts from the Figure 4 counts within AmesNet’s true positives. This isolated the compounds that each TCL variant recovered but its STL counterpart missed as false negatives across the 13 mutagenic substructure classes. TCL-ChemProp recovered 54 more than STL-ChemProp, TCL-RF recovered 27 more than STL-RF, and TCL-GROVER recovered 25 more than STL-GROVER. These gains are consistent with the TCL framework recovering mutagenic toxicophores whose detection depends on bacterial strain and S9 activation assay context.

Even after these TCL gains, AmesNet recovered additional mutagenic compounds that the encoder-swap STL-to-TCL models missed. Across the 13 mutagenic substructure classes, AmesNet recovered 93 more true positives than TCL-ChemProp. TCL-ChemProp was the next-best model in the TCL benchmark. One plausible interpretation is that AmesNet’s advantage reflects both the TCL framework and a molecular representation that encodes mutagenicity-relevant structure differently from state-of-the-art message-passing, graph transformer, and decision tree architectures.

### Operational Utility

#### AmesNet as a Screening Filter

AmesNet is best positioned as an early stage computational screening filter. Triage requires both high sensitivity and retained discriminative ability because false negatives allow mutagenic compounds to advance undetected. AmesNet achieved the highest sensitivity and BA on the Lui et al. benchmark. This positioning suits ultralarge virtual screening workflows where billions of compounds are assessed computationally. Compounds that pass the filter can then proceed to rule-based review at a reduced library size before experimental confirmation. The ICH M7­(R2) framework calls for two complementary methodologies: a statistical QSAR model and an expert rule-based SAR model. AmesNet addresses the statistical component and should be paired with structural-alert methods to satisfy this framework.

#### Sensitivity Must Be Interpreted with Trade-Off Metrics

Reported sensitivity values are difficult to interpret without corresponding trade-off metrics. Published DeepAmes^10^ reports a headline sensitivity of 0.87. The corresponding trade-off metrics in DeepAmes’ additional materials show markedly reduced discrimination: specificity of 0.18, MCC of 0.04, and BA of 0.52. A BA of 0.52 means the model is effectively guessing and provides no reliable predictive signal. In practice, DeepAmes flags the majority of compounds as mutagenic and limits utility for screening triage. DeepAmes’ sensitivity of 0.87 was achieved by sweeping class-weights to maximize sensitivity on the test set (weight = 16). This reflects posthoc operating-point selection and is not directly comparable to a standard held-out test evaluation.

Sensitivity alone overstates screening utility when achieved by broadly calling compounds positive. This reflects an inherent trade-off in binary classification. Tuning a model toward higher sensitivity generally lowers specificity, and the reverse holds as well. AmesNet was therefore evaluated with sensitivity, specificity, PPV, MCC, and BA. On the Lui et al. test set, AmesNet achieved a sensitivity of 0.72, specificity of 0.89, PPV of 0.48, BA of 0.81, and MCC of 0.51. These values show that AmesNet improves mutagen recovery without collapsing specificity or overall discrimination. The PPV represents 3.6× enrichment over the 13% base rate on the Lui et al. test set. This enrichment is consistent with AmesNet’s role as a triage filter that concentrates the positive-suspect set for downstream rule-based and experimental review.

The Foil et al. benchmark (Supporting Information) illustrates why these trade-off metrics are necessary. The STL-DeepAmes reimplementation behaves reasonably on Lui et al. with a sensitivity of 0.69 and BA of 0.76. STL-DeepAmes then degenerates on Foil et al. with a sensitivity of 0.97 accompanied by specificity of 0.05, BA of 0.51, MCC of 0.05, and PPV of 0.31. This pattern indicates near-saturated positive calling rather than useful discrimination and reproduces the failure mode of the published class-weighted configuration under a distinct OOD test set. By contrast, AmesNet retained meaningful sensitivity with substantially stronger specificity, BA, MCC, and PPV. AmesNet therefore provides a more balanced screening profile than models whose sensitivity is driven primarily by excessive positive calls.

#### Applicability Domain and Coverage

Applicability domain remains an important constraint for prospective use. The Lui et al. test set had a mean maximum similarity of 0.50 to the training/validation set. The Foil et al. test set had a mean maximum similarity of 0.47. Coverage decreased as the similarity threshold became more stringent. For the Lui et al. test set, 51%, 70%, and 78% of compounds fell below Tanimoto thresholds of 0.50, 0.60, and 0.65, respectively. For the Foil et al. test set, the corresponding values were 61%, 78%, and 85%. These results show that a large fraction of both benchmarks lies outside conservative similarity-based applicability-domain thresholds. The performance observed on the Lui et al. and Foil et al. (Supporting Information) benchmarks supports AmesNet’s ability to generalize across chemically distant compounds.

### Beyond Ames

Ongoing work will extend this TCL framework into a suite of models for additional ADMET end points used in preclinical development.

## Conclusions

Toxicity assessment remains a prerequisite for advancing novel small-molecule therapeutics to human trials. The Ames test is a core genotoxicity assay for identifying mutagenic risk. Developers often wait to complete Ames studies until a candidate approaches regulatory submission because these experiments are costly and frequently deprioritized relative to bioactivity optimization.

In response, the FDA and international regulatory agencies have issued a clear call to action to operationalize credible *in silico* toxicology approaches that can reduce these late-stage bottlenecks. *In silico* Ames prediction has not reliably mitigated this bottleneck because existing Ames QSAR models do not sustain high sensitivity and discriminative ability under chemical novelty. Optimizing for higher sensitivity has historically forced a trade-off in overall model performance, which can be measured by BA. Low sensitivity is a critical failure because false negatives allow mutagenic compounds to advance undetected and recreate the same late-stage bottleneck these *in silico* approaches are intended to prevent.

AmesNet addresses this sensitivity failure by introducing a TCL modeling paradigm that conditions predictions on assay state variables. These states include strain identity and metabolic activation (±S9). All prior Ames AI models follow the Task-Unconditioned Modeling paradigm and generate predictions without explicit conditioning on assay state (Table 1). AmesNet has an assay-state conditioning channel and a ChemPrint-derived molecular encoder. ChemPrint has shown experimental generalization to chemically novel space in Model Medicines’ antiviral and oncology programs. In this work, sensitivity, BA, and MCC are evaluated on held-out OOD benchmarks designed to measure model performance under chemical novelty.

AmesNet achieves a sensitivity of 0.72 (95% CI: 0.68–0.76) with a BA of 0.81 (95% CI: 0.78–0.83) on the Lui et al. test set, ranking first on both metrics among all evaluated models. AmesNet outperforms a reimplementation of the FDA’s DeepAmes across both benchmarks. On Lui et al., DeepAmes achieves a sensitivity of 0.69 (95% CI: 0.64–0.73) and a BA of 0.76 (95% CI: 0.73–0.78). On Foil et al. (Supporting Information), DeepAmes reaches a sensitivity of 0.97 (95% CI: 0.96–0.98) but collapses to a BA of 0.51 (95% CI: 0.51–0.52) and an MCC of 0.05 (95% CI: 0.03–0.07), reflecting near-saturated positive calling rather than mutagen recognition. By simultaneously improving sensitivity and overall discrimination across the OOD benchmarks, AmesNet overcomes the historical trade-off between sensitivity and overall model performance. Structural enrichment analysis further links these gains to enhanced recognition of historically challenging chemotypes whose mutagenicity depends on specific assay conditions such as strain and metabolic activation.

The performance lift provided by the TCL framework was evaluated by injecting the assay-conditioning channel into message-passing (ChemProp), graph-transformer (GROVER), and Random Forest architectures. On the Lui et al. test set, TCL improves sensitivity, BA, and MCC when injected into message-passing and Random Forest architectures. AmesNet still outperforms every TCL-adjusted encoder on all benchmarks, indicating that AmesNet’s molecular encoder provides a contribution beyond the conditioning channel alone. These results support TCL modeling as a practical advance for reliable *in silico* mutagenicity screening and position AmesNet as a high-confidence early screening tool to reduce late-stage safety attrition risk in modern drug discovery pipelines.

## Limitations

AmesNet was trained and tested on two public data sets that may not capture the full diversity of Ames positive and negative chemistry. The ChemPrint molecular encoder is proprietary and not publicly released. AmesNet may be released in the future. Run-to-run training variance has not been characterized. The confidence intervals reported in this work reflect test-set bootstrapping rather than variability across random initializations.

## Supplementary Material











## Data Availability

All data and code (STL, uMTL, gMTL, TCL encoder swaps, and bootstrapping) are available in the following GitHub repository: https://github.com/Model-Medicines/TCL-Ames

## Notes

Results for Benchmarks 2.1 and 2.2 on the Foil et al. test set, including performance figures and structural enrichment analyses (PDF)
Data sets used to develop and evaluate the models (ZIP)
Applicability domain assessment and label composition for each data set (ZIP)
Test-set prediction scores for each evaluated model (ZIP)
Bootstrap performance metric reports (sensitivity, specificity, BA, MCC, PPV) with 95% confidence intervals and confusion matrices for all evaluated models (ZIP)
