# Supplementary material for: AmesNet: A Task-Conditioned Deep Learning Model with Enhanced Sensitivity and Generalization in Ames Mutagenicity Prediction
Source: Chem Res Toxicol. 2026 Jun 29;39(10):1946–56. doi: 10.1021/acs.chemrestox.6c00082 (PMC13685487; doi:10.1021/acs.chemrestox.6c00082)
Supplement: Supplementary file 1 [file tx6c00082_si_001.pdf]

Supporting Information for: AmesNet: A Task-Conditioned Deep Learning Model with Enhanced Sensitivity and Generalization in Ames Mutagenicity Prediction

Tyler J. Umansky[1], Virgil A. Woods[1], Sean M. Russell[1], and Daniel J. Haders[1*]

[1]Model Medicines, San Diego, California, United States 92122

Results

Benchmark 2.1 (Foil et al.): AmesNet vs. Task-Unconditioned Modeling Paradigms

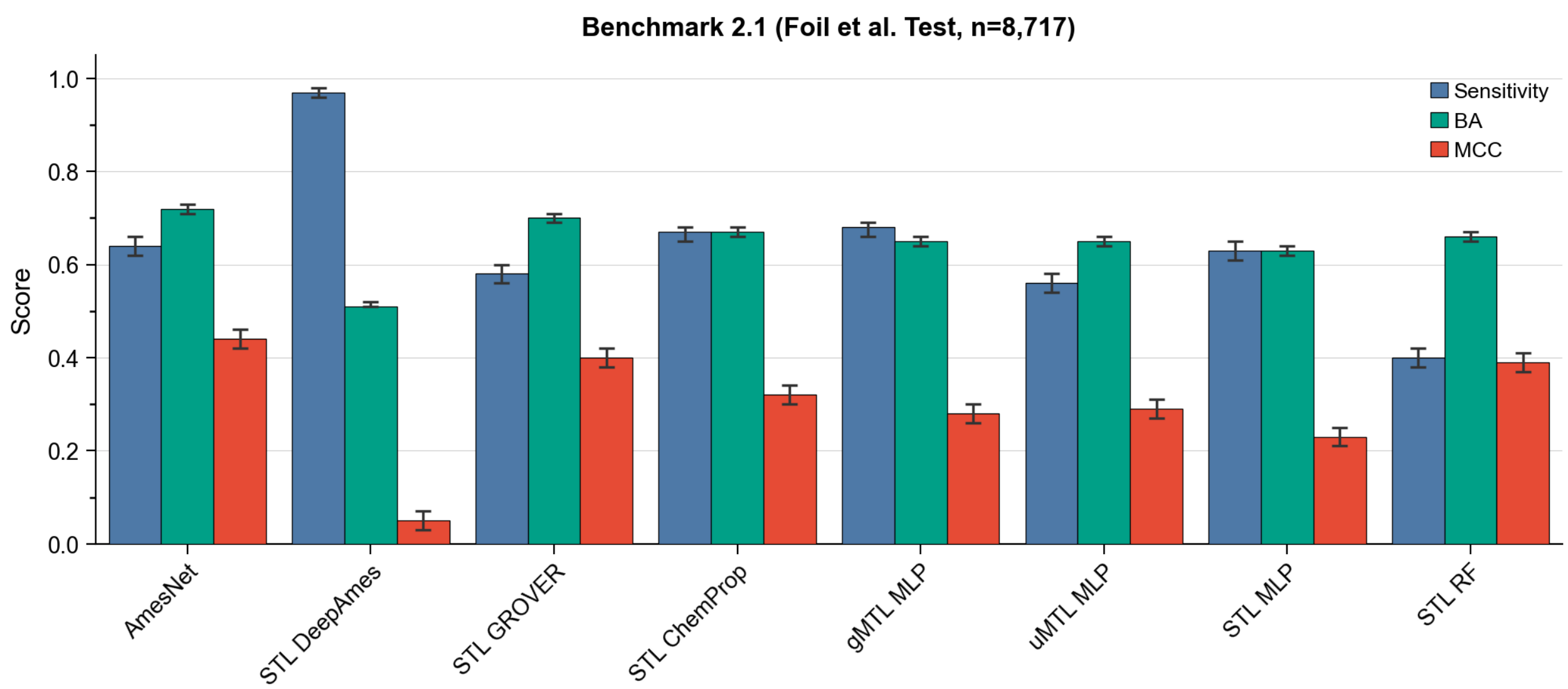

Figure S1: Sensitivity, balanced accuracy, and Matthews correlation coefficient on the Foil et al. test set. Error bars indicate 95% confidence intervals from a stratified bootstrap (n = 1,000) on each model's prediction set.

Benchmark 2.1: Sensitivity on Foil et al. Test Set

Benchmark 2.1 evaluated the Foil et al. test set performance of AmesNet against established Task-Unconditioned Ames QSAR frameworks (STL, uMTL, and gMTL) on sensitivity in an OOD setting. OOD sensitivity and 95% confidence intervals were estimated as described in Methods (Figure S1). AmesNet achieved a sensitivity of 0.64 (95% CI: 0.62–0.66). Within the STL framework, the STL-MLP achieved 0.63 (95% CI: 0.61–0.65), STL-ChemProp achieved 0.67 (95% CI: 0.65–0.68), STL-GROVER achieved 0.58 (95% CI: 0.56–0.60), STL-DeepAmes achieved 0.97 (95% CI: 0.96–0.98), and STL-RF achieved 0.40 (95% CI: 0.38–0.42). Within the uMTL framework, the uMTL-MLP achieved 0.56 (95% CI: 0.54–0.58). Within the gMTL framework, the gMTL-MLP achieved 0.68 (95% CI: 0.66–0.69). AmesNet achieves higher sensitivity than four models, non-inferior sensitivity to two models, and inferiority to one model, as evidenced by non-overlapping 95% bootstrap confidence intervals. AmesNet achieved sensitivity changes of −0.33 (−34%) over

STL-DeepAmes, +0.01 (2%) over STL-MLP, +0.24 (60%) over STL-RF, −0.03 (−4%) over STL-ChemProp, +0.06 (10%) over STL-GROVER, +0.08 (14%) over uMTL-MLP, and −0.04 (−6%) over gMTL-MLP.

Benchmark 2.1: Balanced Accuracy on Foil et al. Test Set

OOD balanced accuracy was calculated with 95% confidence intervals (Figure S1). AmesNet achieved a balanced accuracy of 0.72 (95% CI: 0.71–0.73). AmesNet demonstrates a substantial improvement in balanced accuracy over all models, as evidenced by non-overlapping 95% bootstrap confidence intervals in all but one model. Within the STL framework, the STL-RF achieved 0.66 (95% CI: 0.65–0.67), STL-MLP achieved 0.63 (95% CI: 0.62–0.64), STL-ChemProp achieved 0.67 (95% CI: 0.66–0.68), STL-GROVER achieved 0.70 (95% CI: 0.69–0.71), and STL-DeepAmes achieved 0.51 (95% CI: 0.51–0.52). Within the uMTL framework, the uMTL-MLP achieved 0.65 (95% CI: 0.64–0.66). Within the gMTL framework, the gMTL-MLP achieved 0.65 (95% CI: 0.64–0.66). AmesNet achieved balanced accuracy improvements of +0.06 (9%) over STL-RF, +0.09 (14%) over STL-MLP, +0.05 (7%) over STL-ChemProp, +0.02 (3%) over STL-GROVER, +0.21 (41%) over STL-DeepAmes, +0.07 (11%) over uMTL-MLP, and +0.07 (11%) over gMTL-MLP.

Benchmark 2.1: Matthews Correlation Coefficient on Foil et al. Test Set

OOD Matthews Correlation Coefficient (MCC) was calculated with 95% confidence intervals (Figure S1). AmesNet achieved a MCC of 0.44 (95% CI: 0.42–0.46). Within the STL framework, the STL-RF achieved 0.39 (95% CI: 0.37–0.41), STL-MLP achieved 0.23 (95% CI: 0.21–0.25), STL-ChemProp achieved 0.32 (95% CI: 0.30–0.34), STL-GROVER achieved 0.40 (95% CI: 0.38–0.42), and STL-DeepAmes achieved 0.05 (95% CI: 0.03–0.07). Within the uMTL framework, the uMTL-MLP achieved 0.29 (95% CI: 0.27–0.31). Within the gMTL framework, the gMTL-MLP achieved 0.28 (95% CI: 0.26–0.30). AmesNet demonstrates a substantial improvement in MCC over all models, as evidenced by non-overlapping 95% bootstrap confidence intervals in all but one model. AmesNet achieved MCC changes of +0.39 (780%) over STL-DeepAmes, +0.21 (91%) over STL-MLP, +0.05 (13%) over STL-RF, +0.12 (38%) over STL-ChemProp, +0.04 (10%) over STL-GROVER, +0.15 (52%) over uMTL-MLP, and +0.16 (57%) over gMTL-MLP.

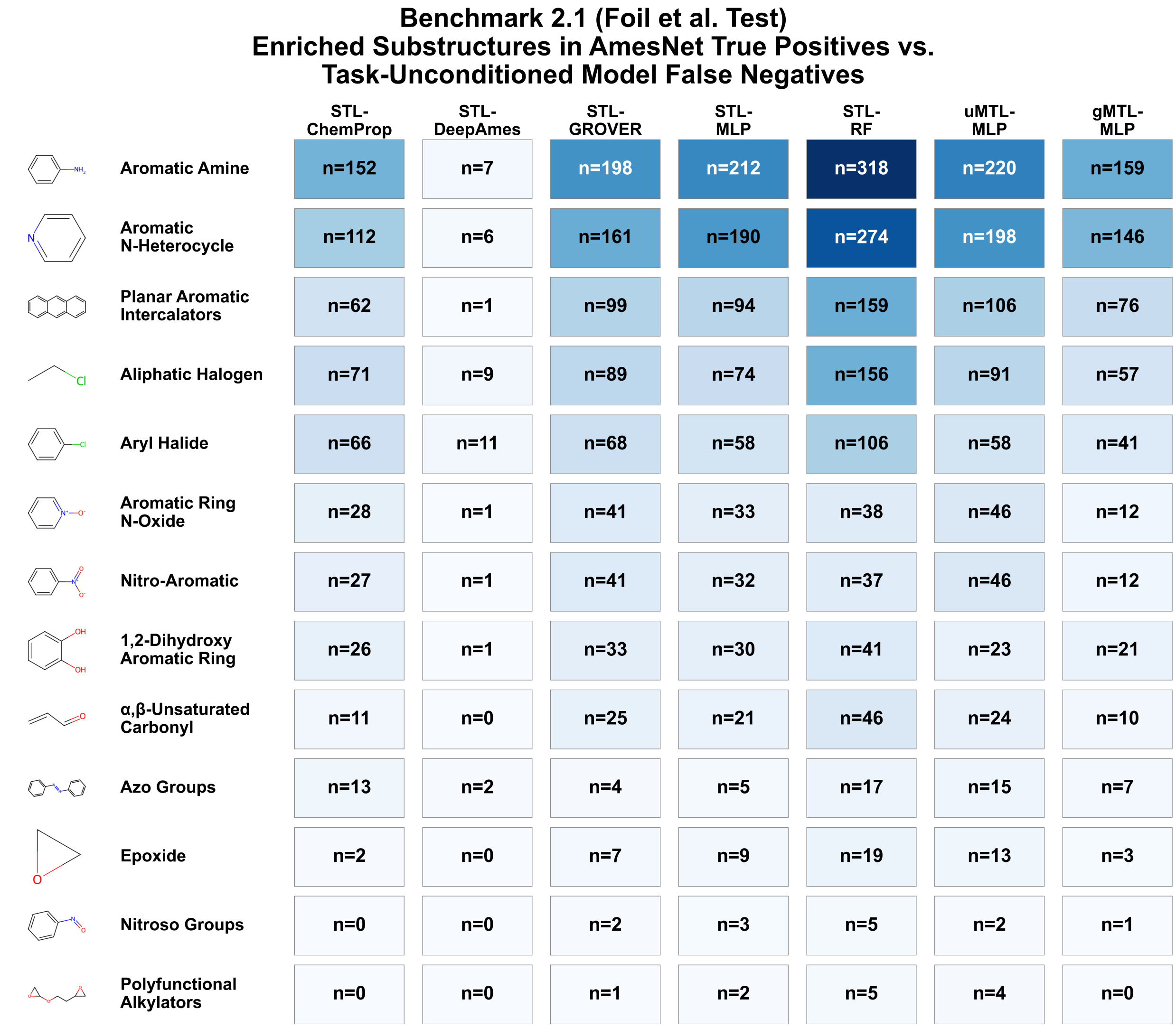

Figure S2: Enriched Substructures in AmesNet True Positives vs. Task-Unconditioned Model False Negatives. Mutagenic substructures observed in compounds correctly predicted by AmesNet but misclassified by unconditioned baseline models (gMTL-MLP, uMTL-MLP, STL-MLP, STL-RF, STL-ChemProp, STL-DeepAmes, STL-GROVER). Values (n) indicate the number of unique substructure-containing compounds where AmesNet achieved true positives while comparison models achieved false negatives. Color intensity encodes n, with darker shades indicating a greater number of substructure-containing compounds.

Benchmark 2.1: Structural Enrichment of True-Positive Predictions in Foil et al. Test Set

Figure S2 summarizes mutagenic substructures present in compounds that AmesNet classified as true positives but that Task-Unconditioned Models misclassified as false negatives when evaluated against the Foil et al. test set. Recurrent categories included aromatic amines (n = 7–318), aromatic N-heterocycles (n = 6–274), planar aromatic intercalators (n = 1–159), aliphatic halogens (n = 9–156), aryl halides (n = 11–106), aromatic ring N-oxides (n = 1–46), nitro-aromatics (n = 1–46), 1,2-dihydroxy aromatic rings (n = 1–41), α,β-unsaturated carbonyls (n = 0–46), azo groups (n = 2–17), epoxides (n = 0–19), nitroso groups (n = 0–5), and polyfunctional alkylators (n = 0–5). These patterns show that false negatives in Task-Unconditioned Models are concentrated in a small set of well-known mutagenic alert classes and that AmesNet correctly identifies many of these cases.

Benchmark 2.2 (Foil et al.): Encoder-Swaps within Task-Conditioning

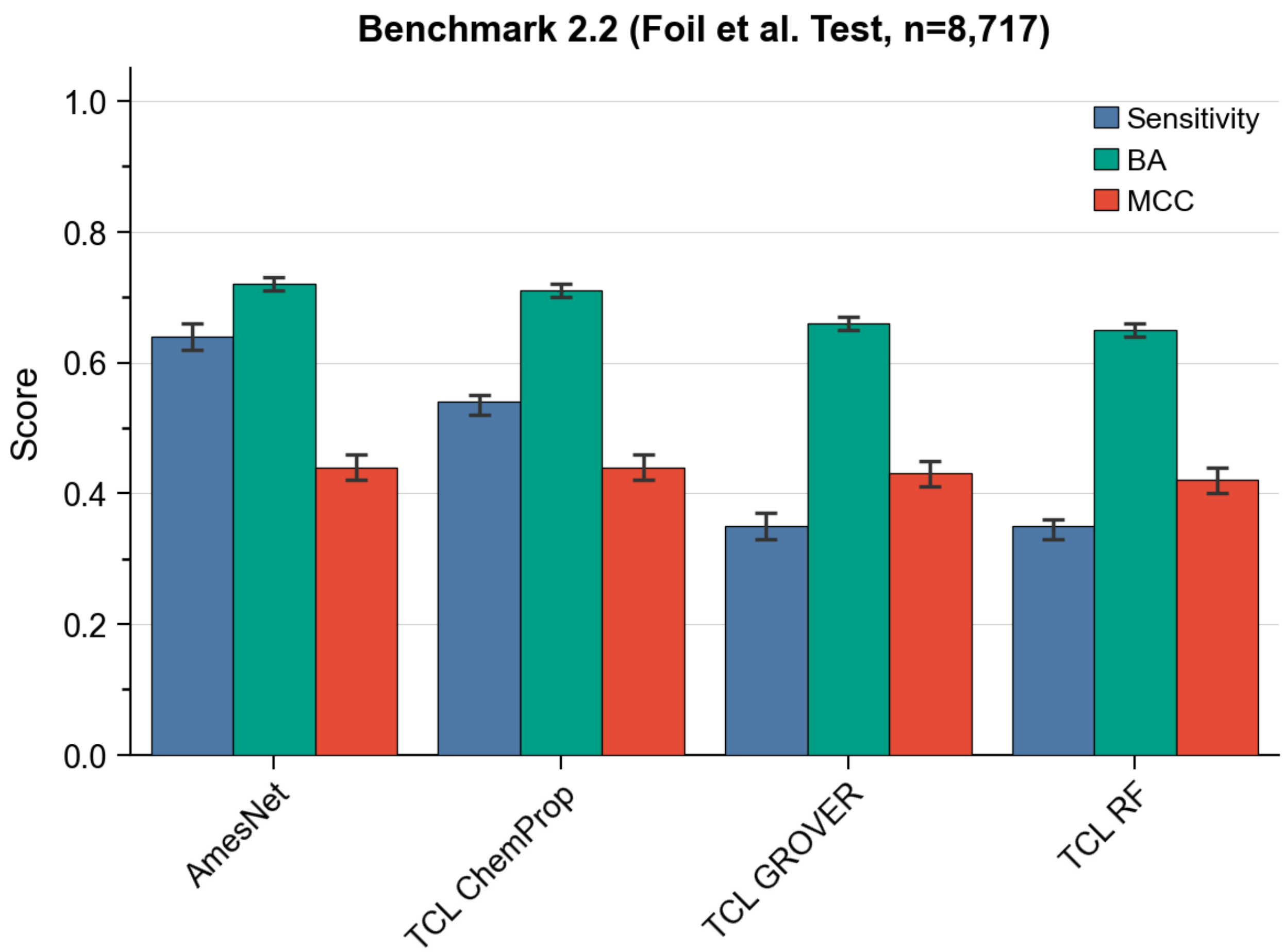

Figure S3: Sensitivity, balanced accuracy, and Matthews correlation coefficient on the Foil et al. test set. Error bars indicate 95% confidence intervals from a stratified bootstrap (n = 1,000) on each model's prediction set.

Benchmark 2.2: Sensitivity on Foil et al. Test Set

AmesNet achieved the highest sensitivity on the Foil et al. test set (Figure S3), reaching a sensitivity of 0.64 (95% CI: 0.62–0.66). TCL-ChemProp decreased sensitivity relative to STL-ChemProp, decreasing from 0.67 (95% CI: 0.65–0.68) to 0.54 (95% CI: 0.52–0.55). TCL-RF decreased sensitivity relative to STL-RF, decreasing from 0.40 (95% CI: 0.38–0.42) to 0.35 (95% CI: 0.33–0.36). TCL-GROVER decreased sensitivity relative to STL-GROVER, decreasing from 0.58 (95% CI: 0.56–0.60) to 0.35 (95% CI: 0.33–0.37).

Benchmark 2.2: Balanced Accuracy on Foil et al. Test Set

Balanced accuracy on the Foil et al. test set was calculated and 95% confidence intervals were reported (Figure S3). AmesNet achieved a balanced accuracy of 0.72 (95% CI: 0.71–0.73). STL-ChemProp achieved 0.67 (95% CI: 0.66–0.68) and TCL-ChemProp achieved 0.71 (95% CI: 0.70–0.72), a +0.04 absolute gain (6% relative). STL-RF achieved 0.66 (95% CI: 0.65–0.67) and TCL-RF achieved 0.65 (95% CI: 0.64–0.66), a −0.01 absolute loss (−2% relative). STL-GROVER achieved 0.70 (95% CI: 0.69–0.71) and TCL-GROVER achieved 0.66 (95% CI: 0.65–0.67), a −0.04 absolute loss (−6% relative). Together, these results show that AmesNet achieves the strongest balanced accuracy and that the TCL framework confers a material balanced accuracy lift for TCL-ChemProp relative to STL-ChemProp.

Benchmark 2.2: Matthews Correlation Coefficient on Foil et al. Test Set

MCC on the OOD test set was calculated and 95% confidence intervals were reported (Figure S3). AmesNet achieved a MCC of 0.44 (95% CI: 0.42–0.46). STL-ChemProp achieved 0.32 (95% CI: 0.30–0.34) and TCL-ChemProp achieved 0.44 (95% CI: 0.42–0.46), a +0.12 absolute gain (38% relative). STL-RF achieved 0.39 (95% CI: 0.37–0.41) and TCL-RF achieved 0.42 (95% CI: 0.40–0.44), a +0.03 absolute gain (8% relative). STL-GROVER achieved 0.40 (95% CI: 0.38–0.42) and TCL-GROVER achieved 0.43 (95% CI: 0.41–0.45), a +0.03 absolute gain (7% relative). Together, these results show that the TCL framework confers a MCC lift for all models.

Benchmark 2.2 (Foil et al. Test)
Enriched Substructures in AmesNet True Positives vs. Encoder-Swap Model False Negatives

Structure	Substructure	TCL-ChemProp	TCL-GROVER	TCL-RF
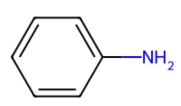	Aromatic Amine	n=178	n=370	n=363
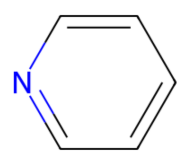	Aromatic N-Heterocycle	n=137	n=281	n=289
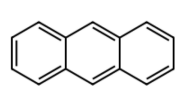	Planar Aromatic Intercalators	n=80	n=205	n=181
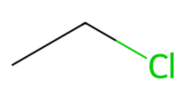	Aliphatic Halogen	n=99	n=169	n=180
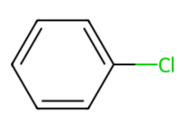	Aryl Halide	n=69	n=122	n=106
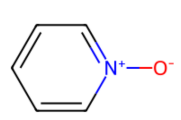	Aromatic Ring N-Oxide	n=49	n=106	n=49
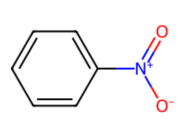	Nitro-Aromatic	n=50	n=106	n=49
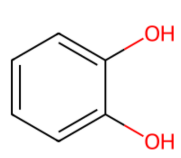	1,2-Dihydroxy Aromatic Ring	n=25	n=45	n=43
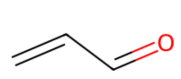	α,β-Unsaturated Carbonyl	n=25	n=48	n=50
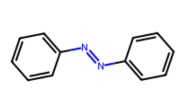	Azo Groups	n=29	n=34	n=35
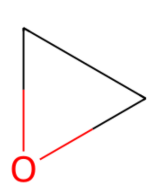	Epoxide	n=8	n=21	n=23
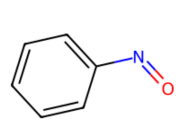	Nitroso Groups	n=2	n=6	n=8
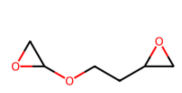	Polyfunctional Alkylators	n=2	n=3	n=6

Figure S4: Enriched Substructures in AmesNet True Positives vs. Encoder-Swap Model False Negatives. Mutagenic substructures observed in compounds correctly predicted by AmesNet but misclassified by encoder-swap TCL models (TCL-ChemProp, TCL-GROVER, TCL-RF). Values (n) indicate the number of unique substructure-containing compounds where AmesNet achieved true positives while comparison models achieved false negatives. Color intensity encodes n, with darker shades indicating a greater number of substructure-containing compounds.

Benchmark 2.2: Structural Enrichment of True-Positive Predictions in Foil et al. Test Set

Structural enrichment analysis (Figure S4) highlights mutagenic substructures that AmesNet recovers as true positives but that TCL-ChemProp, TCL-GROVER, and TCL-RF miss as false negatives within the Foil et al. test set. Recurrently missed classes were aromatic amines (n = 178–370), aromatic N-heterocycles (n = 137–289), planar aromatic intercalators (n = 80–205), aliphatic halogens (n = 99–180), aryl halides (n = 69–122), aromatic ring N-oxides (n = 49–106), nitro-aromatics (n = 49–106), 1,2-dihydroxy aromatic rings (n = 25–45), α,β-unsaturated carbonyls (n = 25–50), azo groups (n = 29–35), epoxides (n = 8–23), nitroso groups (n = 2–8), and polyfunctional alkylators (n = 2–6). Relative to STL-ChemProp, TCL-ChemProp does not show broad recovery for aromatic amines (152 to 178), aromatic N-heterocycles (112 to 137), planar aromatic intercalators (62 to 80), aliphatic halogens (71 to 99), aryl halides (66 to 69), aromatic ring N-oxides (28 to 49), nitro-aromatics (27 to 50), 1,2-dihydroxy aromatic rings (26 to 25), α,β-unsaturated carbonyls (11 to 25), azo groups (13 to 29), epoxides (2 to 8), nitroso groups (0 to 2), and polyfunctional alkylators (0 to 2). Relative to STL-RF, TCL-RF does not show broad recovery for aromatic amines (318 to 363), aromatic N-heterocycles (274 to 289), planar aromatic intercalators (159 to 181), aliphatic halogens (156 to 180), aryl halides (106 to 106), aromatic ring N-oxides (38 to 49), nitro-aromatics (37 to 49), 1,2-dihydroxy aromatic rings (41 to 43), α,β-unsaturated carbonyls (46 to 50), azo groups (17 to 35), epoxides (19 to 23), nitroso groups (5 to 8), and polyfunctional alkylators (5 to 6). Recovery from STL-GROVER to TCL-GROVER does not show broad recovery for aromatic amines (198 to 370), aromatic N-heterocycles (161 to 281), planar aromatic intercalators (99 to 205), aliphatic halogens (89 to 169), aryl halides (68 to 122), aromatic ring N-oxides (41 to 106), nitro-aromatics (41 to 106), 1,2-dihydroxy aromatic rings (33 to 45), α,β-unsaturated carbonyls (25 to 48), azo groups (4 to 34), epoxides (7 to 21), nitroso groups (2 to 6), and polyfunctional alkylators (1 to 3). Together, these patterns indicate that within the TCL paradigm, AmesNet's molecular representation more reliably recognizes mutagenic alert classes than TCL-ChemProp, TCL-GROVER, and TCL-RF.
